# Predicting the time to detect moderately virulent African swine fever virus in finisher swine herds using a stochastic disease transmission model

**DOI:** 10.1186/s12917-022-03188-6

**Published:** 2022-03-02

**Authors:** Sasidhar Malladi, Amos Ssematimba, Peter J. Bonney, Kaitlyn M. St. Charles, Timothy Boyer, Timothy Goldsmith, Emily Walz, Carol J. Cardona, Marie R. Culhane

**Affiliations:** 1 Secure Food Systems Team, University of Minnesota, Saint Paul, MN 55108 USA; 2 Department of Mathematics, Faculty of Science, Gulu University, Gulu, Uganda; 3 Center for Epidemiology and Animal Health, Veterinary Services, Animal and Plant Health Inspection Service, United States Department of Agriculture, Fort Collins, Colorado USA

**Keywords:** Surveillance, Mortality triggers, Modeling, Clinical signs detection, African Swine Fever, Moderately virulent strain

## Abstract

**Background:**

African swine fever (ASF) is a highly contagious and devastating pig disease that has caused extensive global economic losses. Understanding ASF virus (ASFV) transmission dynamics within a herd is necessary in order to prepare for and respond to an outbreak in the United States. Although the transmission parameters for the highly virulent ASF strains have been estimated in several articles, there are relatively few studies focused on moderately virulent strains. Using an approximate Bayesian computation algorithm in conjunction with Monte Carlo simulation, we have estimated the adequate contact rate for moderately virulent ASFV strains and determined the statistical distributions for the durations of mild and severe clinical signs using individual, pig-level data. A discrete individual based disease transmission model was then used to estimate the time to detect ASF infection based on increased mild clinical signs, severe clinical signs, or daily mortality.

**Results:**

Our results indicate that it may take two weeks or longer to detect ASF in a finisher swine herd via mild clinical signs or increased mortality beyond levels expected in routine production. A key factor contributing to the extended time to detect ASF in a herd is the fairly long latently infected period for an individual pig (mean 4.5, 95% P.I., 2.4 - 7.2 days).

**Conclusion:**

These transmission model parameter estimates and estimated time to detection via clinical signs provide valuable information that can be used not only to support emergency preparedness but also to inform other simulation models of evaluating regional disease spread.

## Introduction

African swine fever (ASF) is a highly contagious pig disease caused by ASF virus (ASFV), a large, enveloped double stranded DNA virus from the genus Asfivirus. The disease is endemic in sub-Saharan Africa and was first described by Montgomery in Kenya in 1921 [1]. Starting from an outbreak of genotype II ASFV in Georgia in 2007, the virus spread widely and affected several countries in eastern Europe and Russia [2]. Genotype II ASFV strains were subsequently introduced into China in 2018 devasting swine populations in several Asian countries and causing extensive economic losses [3].

The highly virulent form of the disease is characterized by high fever, anorexia, recumbency and severe disseminated hemorrhage with mortality rates approaching 100 percent [4, 5]. Moderately virulent strains produce lower mortality and less severe clinical signs such as reduced feed intake, lethargy, and reddening of the skin [6]. The clinical presentations of pigs infected with highly virulent or moderately virulent strains and the duration of the peracute, acute, subacute, and chronic disease states have been reviewed by Sanchez-Vizcaino et al., [7]. Although high fever and loss of appetite are typical during the peracute and acute phases of ASF infection regardless of strain virulence, the other clinical signs are quite variable [4]. Furthermore, the subacute and chronic phases of disease are usually only attributed to moderately virulent strains [7]. A sizable fraction of the pigs may recover from clinical disease caused by moderately virulent ASF strains but remain viremic for a long duration [8]. Although genotype II ASFV strains are typically highly virulent, some recent studies of strains from Estonia suggest that circulating viruses have evolved into moderately virulent strains [2] resulting in a decrease in severity of clinical disease observed, more comparable to the ASF outbreaks in Spain and Portugal in the 1960s [9].

Understanding ASF transmission dynamics within a herd is important to inform several aspects of emergency preparedness and response such as surveillance design, business continuity planning and development of effective outbreak control strategies. Although several articles have estimated transmission parameters for the highly virulent genotype ASFV strains, relatively few studies focused on moderately virulent strains [10, 11]. Considering moderately virulent strains in emergency preparedness activities is important to develop robust plans given the possibility that highly virulent strains may evolve into moderately virulent strains overtime. de Carvalho Ferreira *et al.* estimated the adequate contact rate (transmission parameter) and the infectious period for two moderately virulent strains of ASFV, Malta 78 and Netherlands 86 [8]. However, additional transmission model parameters such as the time to onset and duration of various types of clinical signs and latently infected period were not estimated in that study. These parameters are essential to estimate the time to detection via observation of clinical signs, increased mortality, and diagnostic testing targeting sampling of the sick and dead pigs.

To obtain these essential parameters, we performed this study in which we estimated the transmission parameters for moderately virulent ASFV strains using data from de Carvalho Ferreira *et al.* (2013). Separate infectious period durations were estimated for pigs that die in the acute disease phase and those that recover from clinical disease and shed over a longer duration. We then used a discrete individual based simulation model to estimate the time to detect ASF infection in a herd based on increased mortality or via presence of clinical signs. The estimated parameters and the predicted time to detect ASFV via clinical signs are useful for informing outbreak response planning, surveillance evaluation and for estimating inputs for regional disease spread models.

## Materials and Methods

We estimated disease state durations using experimental data for moderately virulent strains Malta 78 and Netherlands 86 used to inoculate pigs in de Carvalho Ferreira et al. [8]. Disease state durations, e.g., latently infected period, the infectious period for pigs that die, and the infectious period for those that recover, were assumed to be Gamma distributed and estimated using a maximum likelihood estimation approach. The likelihood expression accounted for censored data where pigs were monitored at daily intervals. Data from all three tables in de Carvalho Ferreira *et al. *were combined to enhance statistical power. For estimating the duration of pig level latent and infectious periods, we used the criterion of qPCR detected equivalent virus titer ≥1.92 log TCID_50_ in oropharyngeal swab to classify a pig as infectious (see criterion 2 in de Carvalho Ferreira *et al.,* [8]). The parameters for the estimated disease state durations are summarized in Table 1. Further details on the estimation of disease state durations are provided in the supplement.

**Table 1:** Disease state duration parameter estimates for moderately virulent ASF strains based on data from de Carvalho Ferreira et al. (2013)

**Parameter**	**Distribution details**	**Value**
**Latently infected period**	Gamma(shape=13.299, scale=0.3384482)	4.501(95% P.I.,2.417,7.223) days
**Infectious period for recovered**	Gamma(shape=55.42012, scale= 0.7950162)	44.06(95% P.I.,33.23,56.394) days
**Infectious period for dead**	Gamma(shape =9.632, scale =0.862)	8.306(95% P.I.,3.918,14.314) days
**Time to onset of severe clinical signs**	Gamma(shape =41.969, scale =0.259)	10.868(95% P.I,7.832,14.394) days
**Time to onset of mild clinical signs**	Gamma(shape =26.257, scale =0.214)	5.614(95% P.I.,3.675,7.956) days
**Duration of mild clinical signs**	Gamma(shape =3.418,scale =3.2)	10.936(95% P.I.,2.58,25.212) days
**Duration of severe clinical signs (severe)**	Gamma(shape =1.027, scale =6.515)	6.694(95% P.I.,0.184,24.408) days
**Fraction of pigs with severe clinical signs**	Beta (22,10)	0.688 (95 % P.I., 0.52,0.833)
**Fraction of pigs dying due ASF**	Beta (13,19)	0.407(95 % P.I., 0.24,0.57)

The timing of onset of clinical signs and their durations varied considerably. The clinical signs were categorized as mild or severe (Table 2 in de Carvalho Ferreira *et al.,* 2012) [12]. Specifically, pigs with any of the following symptoms were considered to have severe clinical signs: neurological signs, labored respiration, vomiting, skin discoloration at level 2 or 3, diarrhea at level 3, body shape at level 2 or 3, or any bloody discharge. Pigs with other signs (e.g., reduced liveliness, loss of appetite, red skin) excluding the severe clinical signs were regarded as having mild clinical signs. The estimated disease state durations and associated parameters are summarized in Table 1.

**Table 2:** Transmission parameter estimates from ASFV experimental data sets provided in de Carvalho Ferreira et al. (2013)

**Experimental data set**	**Adequate contact rate Mode, (95% C.I.)**
Malta 78 ASFV low dose (Table 2 in de Carvalho Ferreira *et al.,* 2013)	3.25 (0.90-9.14)
Malta 78 ASFV high dose (Table 3 in de Carvalho Ferreira *et al.,* 2013)	8.63 (2.71-14.63)
Netherlands 86 ASFV (Table 4 in de Carvalho Ferreira *et al.,* 2013)	1.2 (0.52-2.33)
Malta 78 ASFV high and low dose experiments	3.15 (1.8-10.62)
Malta 78 ASFV high and low dose and Netherlands 86 ASF combined (Tables 2-4 de Carvalho Ferreira *et al.,* 2013)	1.64 (1-2.74)

We used an approximate Bayesian computation algorithm to estimate the adequate contact rate from the experimental data sets in de Carvalho Ferreira *et al.* (2013). In this algorithm, the adequate contact rate (β) values in each iteration were sampled from a wide uniform prior (0.2-15 per day). Iterations where the simulated times of infection and time to onset of infectiousness among the contact pigs match the experimental data were selected to obtain the posterior distribution. The contact rates were estimated for the individual data sets both separately and jointly, combining all three data sets in alternative estimation scenarios. The parameter estimation algorithm was coded in R and C and the library Rmpi was used to implement the code under a parallel computing framework. Details of the transmission parameter estimation are provided in the supplement.

We used a stochastic individual based transmission model to simulate ASF spread within a population (e.g., a barn), and predict the time to detection via clinical signs. Similar to other ASF transmission studies, we assumed that transmission was frequency dependent [13]. Given this formulation, the number of newly infected pigs in a period *C*_*(t)*_ is binomially distributed with probability *P*_*new(t)*_

$${P}_{new(t)} =1-Exp\left(\frac{-\beta I(t)}{N(t)}\right)$$

### Eq. 1

Here N(t) is the total number of pigs in the population, I(t) is the number of infectious pigs in the population, and *β* is the adequate contact rate. The model simulates the number of pigs in Susceptible (S), Latent (E), Infectious (I) and recovered(R) or dead (D) states in 0.01 day time steps. The disease state durations were all modeled to be Gamma distributed. An infectious pig may transition to the dead state with a probability *P*_*mort*_ or transition to recovered state otherwise. Regardless of the pig’s transition to the dead or recovered state, the pig remained infectious with separate infectious period distributions used for pigs that recovered versus pigs that died. This is consistent with the available experimental data which indicated a much shorter infectious period for pigs that died. Clinical signs were modeled as an additional attribute for an infectious pig or a non-infectious pig that has recovered and is no longer shedding virus. We modeled that only a proportion of infected pigs exhibit one or more severe clinical signs depending on the probability *P*_*clin*_.

ASF detection via increased mortality was based on the timing of when the mortality exceeded levels expected in routine production triggering further diagnostic investigation. The time to detection via increased mortality was calculated as the first day post exposure when the simulated daily mortality exceeded a specified fraction of the herd (i.e., the first day the mortality trigger threshold was reached). To estimate the time to detection, we superimposed the daily disease mortality predictions from the transmission model with predictions of normal mortality unrelated to ASFV. The time to detection was estimated under two contact rate scenarios, baseline and slow. The baseline scenario used a contact rate of 3.15 (1.80 - 10.62) per day based on the combined estimate for Malta 78 ASFV while a contact rate of 1.64 (1.00 - 2.74) per day was used in the slow scenario. Normal mortality associated with routine production causes unrelated to ASFV was simulated as an empirical distribution based on weekly mortality data for 248 pig herds from 4 pig farming systems in North America. The mean weekly mortality fraction in the pig production data was 0.00331 (95% P.I., 0, 0.013). In each simulation iteration, weekly mortality fractions for the required number of weeks were obtained from a randomly selected herd. The variability in daily mortality within each week was then simulated as a Poisson distribution with mean equal to herd size multiplied by weekly mortality fraction divided by seven. A Pert distribution with minimum, most likely and maximum values of 1000, 2496, 5000 pigs was used for the number of swine in the population representative of large finisher swine operations in Minnesota [14].

The time to detection via morbidity was calculated as the first day post exposure when the number of pigs with mild (or severe) clinical signs exceeded a specified percent of the herd (i.e., the first day the morbidity trigger threshold for mild (or severe) clinical signs was reached). Robust data on the frequency of clinical signs due to other production or disease issues unrelated to ASFV was not available. Therefore, we simulated the fraction of pigs with mild clinical signs using a Pert (0.0025, 0.005, 0.04) distribution based on North American swine industry expert opinion. Severe clinical signs due to causes other than ASFV were not simulated as they would be infrequent under routine production.

We performed 10,000 iterations of the ASF within herd disease transmission model coded in the programming languages R and C as described above to predict the time to detection under various trigger thresholds for mild clinical signs, severe clinical signs, and increased mortality. The mean time to detection and the two sided 90 percent prediction interval were calculated directly based on the simulation results for the 10,000 iterations. False trigger rates for various daily and weekly mortality thresholds were estimated from 10,000 iterations of simulated normal mortality using routine production data as described above.

## Results

The estimated disease state durations are provided in Table 1. We observe that the time to onset of mild clinical signs, 5.61 (95% P.I., 3.68 - 7.96) days, is comparable to the latently infected period of 4.5 (95% P.I., 2.42 - 7.20) days. Not surprisingly, severe clinical signs occurred much later in the disease course relative to mild clinical signs. In addition, there was a greater variability associated with severe clinical signs with only a fraction of the exposed pigs having these severe clinical signs. The transmission parameter estimates varied between different experimental data sets with the Netherlands 86 ASFV strain having a lower contact rate indicative of slower spread (Table 2).

**Fig. 1:**
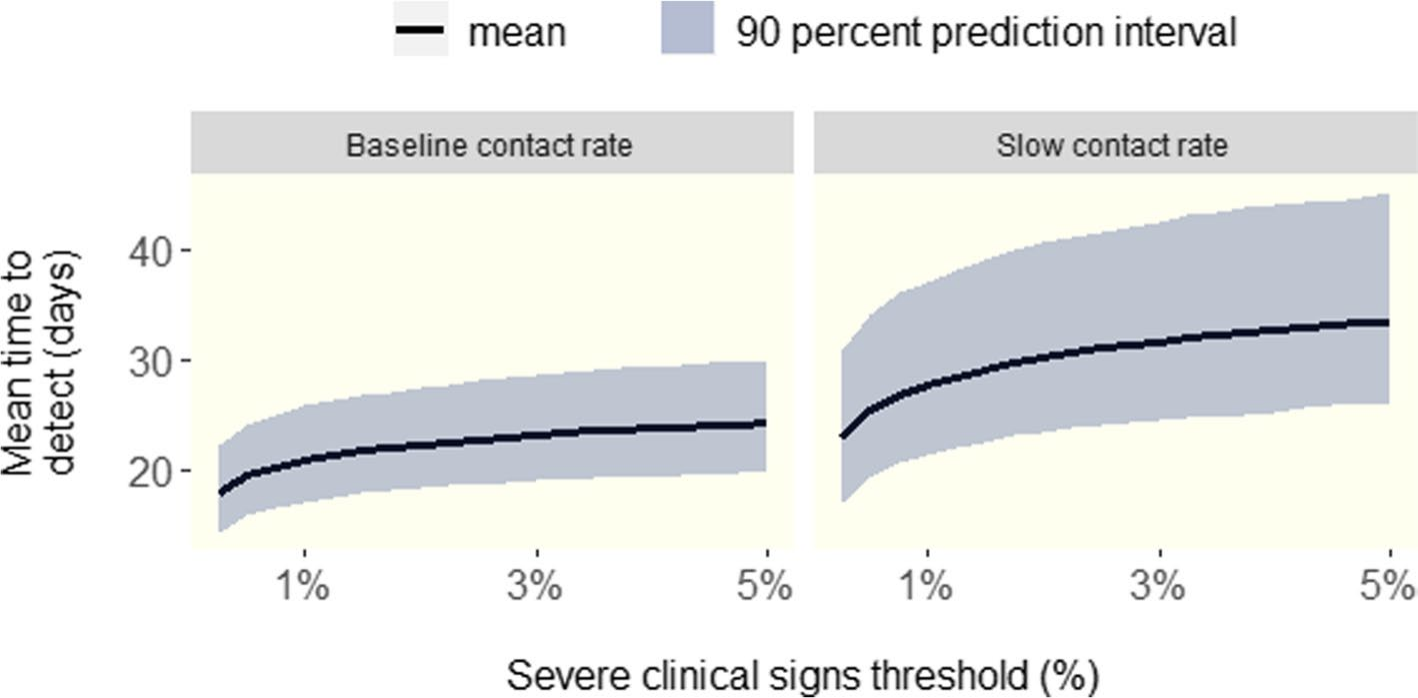
Time to detect ASF in a large finisher herd using various morbidity trigger thresholds for severe clinical signs under the baseline and slow adequate contact rate scenarios

The predicted time to detection given various morbidity trigger threshold levels for severe clinical signs above is shown in Fig. 1. The simulation results indicated that it may take two weeks or more post-exposure of the herd to detect ASF via severe clinical signs. For instance the predicted time to detection via severe clinical signs using a 1% morbidity trigger threshold was 21 (95% P.I., 17 - 26) days for the baseline contact rate scenario and 28 (95% P.I., 21 - 37) days under the slow contact scenario.

**Fig. 2:**
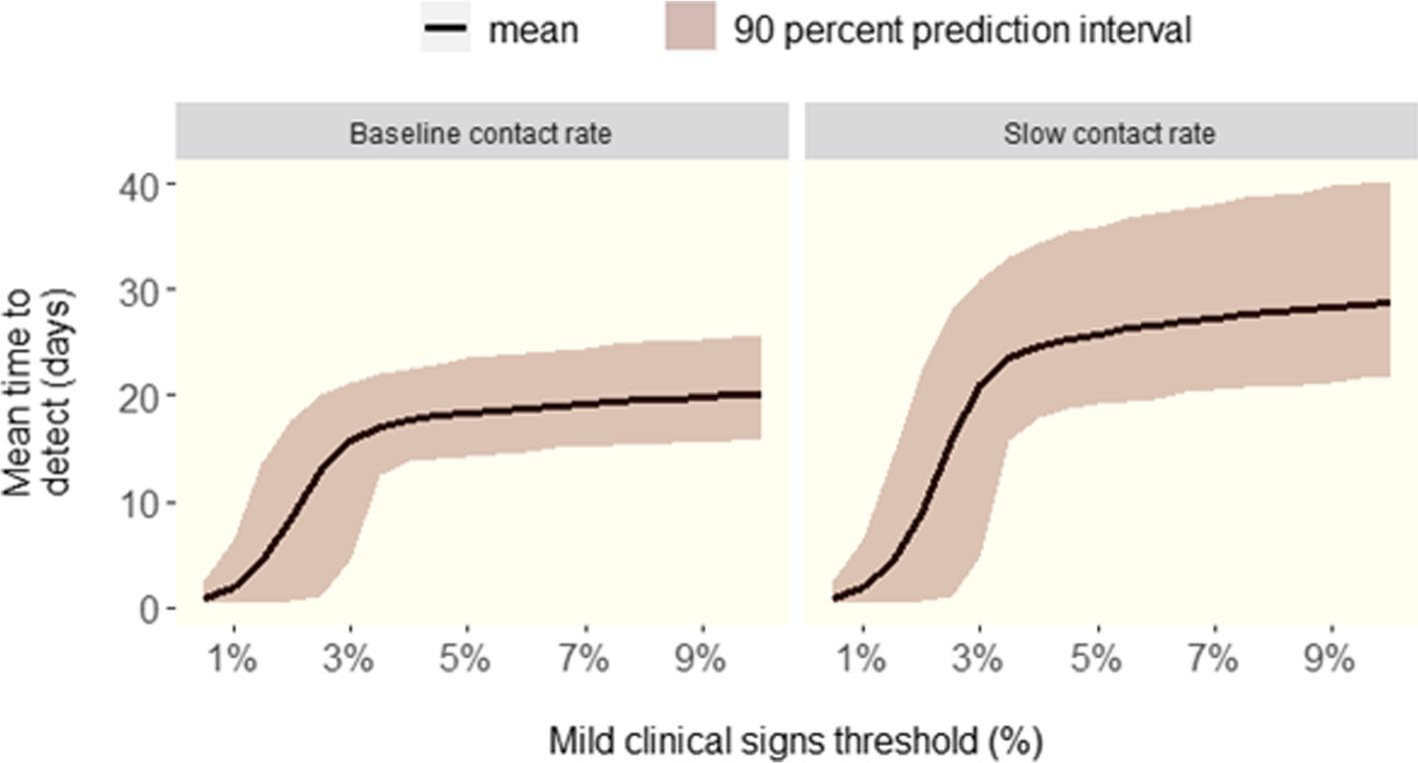
Time to detect ASF in a large finisher herd using various morbidity trigger thresholds for mild clinical signs under the baseline and slow adequate contact rate scenarios

The predicted time to detect ASF in finisher herds via mild clinical signs is shown in Fig. 2. At a conservative morbidity trigger threshold of 9% of the herd on a day, the predicted time to detection via mild clinical signs was 20 (95% P.I., 16 - 25) and 28 (95% P.I., 21 - 40) days under the baseline and slow contact rates respectively. The time to detection decreases rapidly for morbidity trigger thresholds less than 2% as many mild clinical signs are nonspecific and may occur due to other reasons in routine pig farm production.

**Fig. 3:**
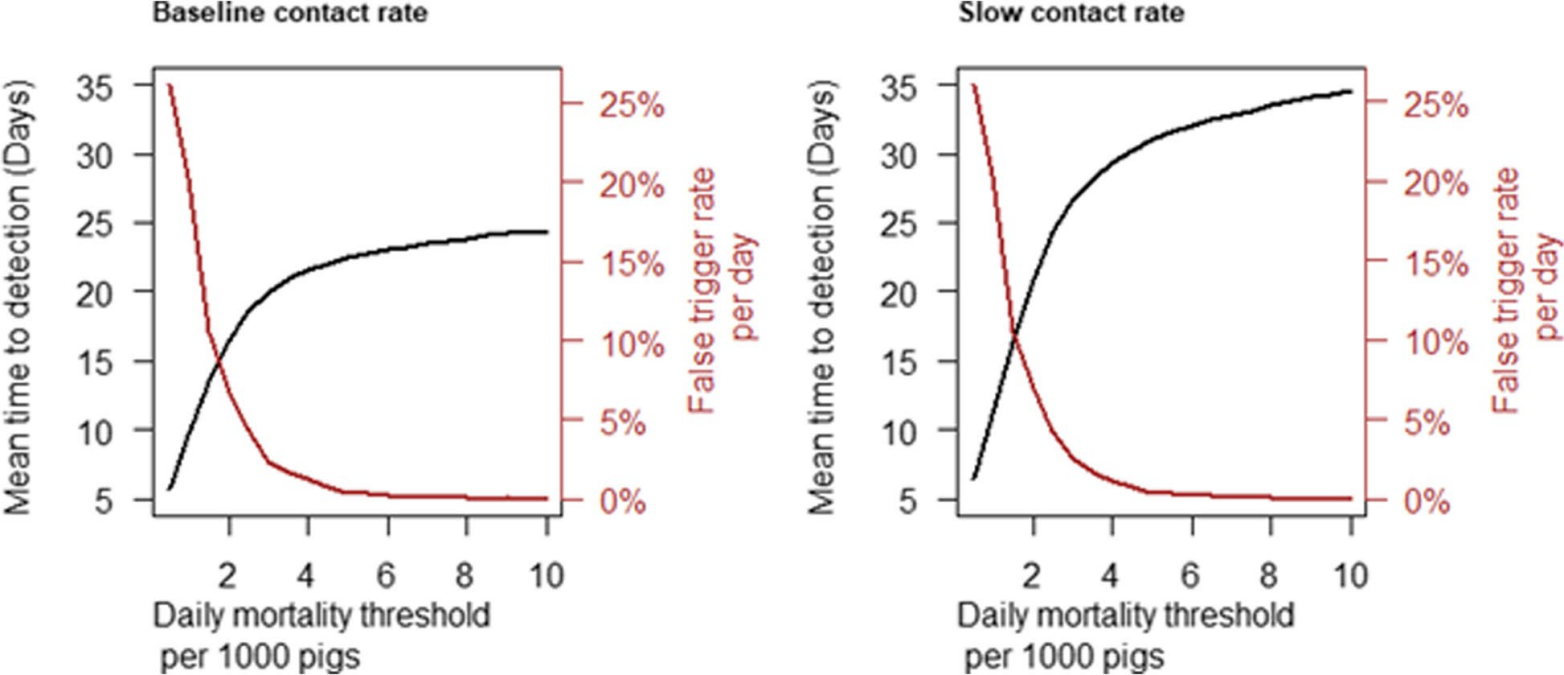
Time to detect ASF in a large finisher herd via increased daily mortality and associated false trigger rate under the baseline adequate contact rate scenario (**A**) and slow adequate contact rate scenario (**B**)

Figure 3 shows the mean time to detection via an absolute daily mortality trigger threshold and the corresponding false trigger rate estimated from routine production data unrelated to ASF. The false trigger rate at a threshold of 0.005 was 0.47%. Given this daily mortality trigger threshold , the predicted time to detection was 22 (95% P.I., 17 - 28) days and 31 (95% P.I., 22 - 43) under the baseline and slow contact rate scenarios.

## Discussion

In this paper, we estimated disease state durations and transmission parameters from data presented in de Carvalho Ferreira *et al.* (2013) to update ASF within herd transmission models used to inform surveillance and risk assessment. The estimated parameters were used in an individual based stochastic disease transmission model to predict the time to detect ASF in a herd via increased morbidity or mortality. Although several studies have modeled the transmission dynamics of highly virulent genotype ASFV strains, relatively few studies focused on moderately virulent strains. Our modeling approach helps capture the transmission dynamics of moderately virulent ASFV strains more accurately by incorporating separate disease state durations for pigs that recover and those that die due to ASF. Although de Carvalho Ferreira *et al.* (2013) provided transmission parameter and infectious period estimates, other simulation model parameters, such as statistical distributions for the time to onset of clinical signs and latently infected period, were not estimated previously [8]. The parameter estimates from the current study enable predicting the time to detect ASFV via observation of clinical signs and inform input parameters for designing active surveillance protocols with targeted sampling of sick and dead pigs. The results on the predicted time to detection are also beneficial to inform between premises simulation models used for evaluating regional outcomes and optimize outbreak response strategies.

Our simulation results indicate that it may take more than two weeks post-exposure to detect moderately virulent ASF under most mortality or morbidity trigger thresholds evaluated. One of the factors contributing to an extended time to detection is the relatively long latently infected period at the individual pig level (mean 4.5 days), which results in relatively slower transmission during the initial stages of the herd infection. For instance, only 2.3 (95% P.I., 1 - 8) pigs were infectious and 1.1 (95% P.I., 1 - 3) pigs had mild clinical signs at 8 days post exposure under the baseline scenario. Nonetheless, ASF infection in the herd was detected via clinical signs and increased mortality in almost all of the simulation iterations. This is because the force of infection and the incidence eventually picks up as the number of infectious pigs increases resulting in rapidly increasing morbidity and mortality during an exponential transmission phase. The results on the predicted time to detection are beneficial to inform emergency preparedness and response plans that provide guidance on disease response strategies and science- and risk-based approaches to facilitate continuity of business [15]. For example, the USDA Highly Pathogenic Avian Influenza response plan specifies daily mortality thresholds in the case definitions of illness compatible with H5/H7 AI infection. The case definitions are used to designate an operation as a suspect premises and initiate further diagnostic investigation as well as other response steps [16]. The predicted time to detection also informs between premises transmission models used to evaluate regional epidemiological outcomes and for evaluating alternative response strategies. In addition, the results provide baseline predictions to evaluate the relative performance of additional active or passive surveillance protocols.

**Fig. 4:**
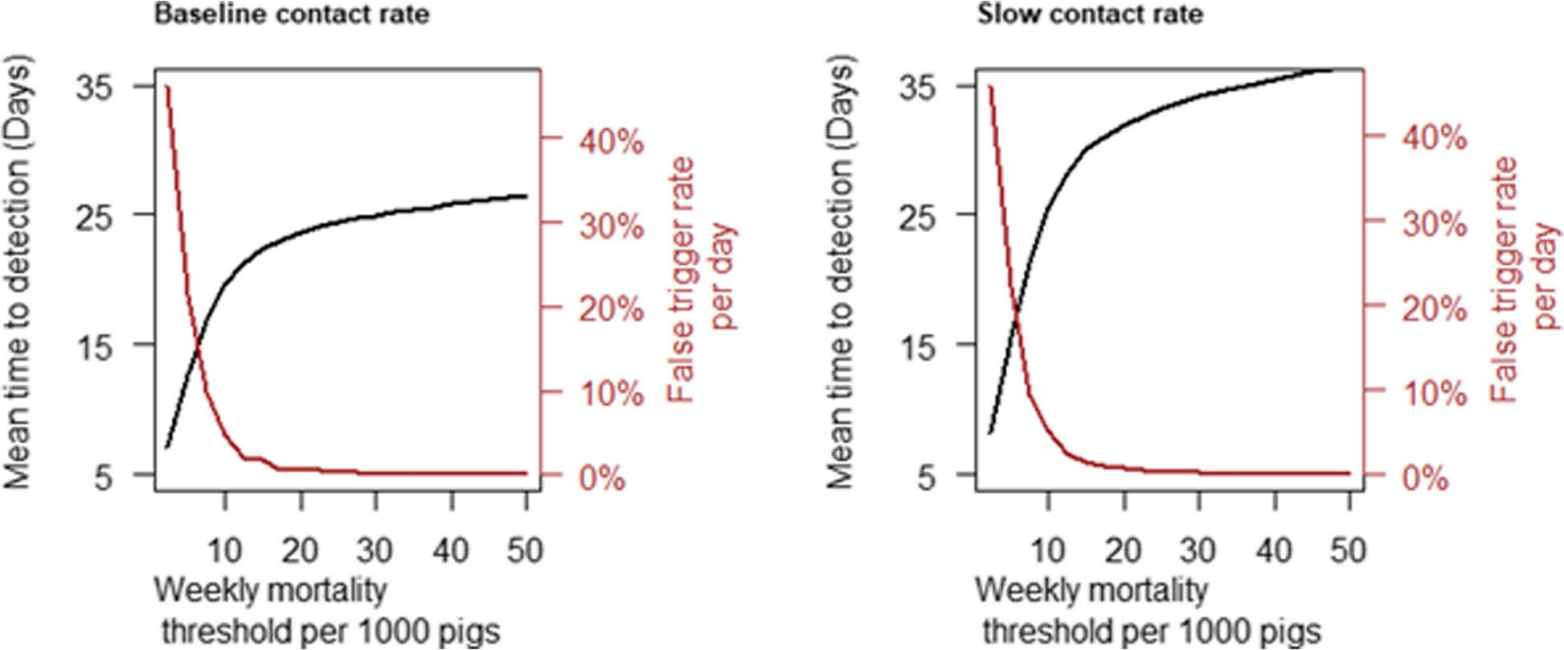
Time to detect ASF in a large finisher herd via increased weekly mortality and associated false trigger rate under the baseline adequate contact rate scenario (**A**) and slow adequate contact rate scenario (**B**)

Risk managers would need to consider the trade-off between earlier detection and excessive false triggers while choosing the appropriate morbidity and mortality triggers for detection via daily numbers of dead pigs or pigs with clinical signs of disease. The results for the daily mortality trigger in Fig. 3 show a rapid increase in the false trigger rate as the threshold was reduced below 4 dead per 1000 pigs in both the baseline and slow spread scenarios. For example, the false trigger rate increased from 0.5% to 1.3% when the daily mortality trigger threshold was decreased from 5 per 1000 to 4 per 1000 pigs. A similar relationship can be observed in the weekly mortality trigger results given in Fig. 4 with the inflection point occurring at a trigger threshold of about 15 per 1000.

We used swine industry expert opinion to estimate the frequency of mild clinical signs as production data on this aspect were not available. Swine industry experts indicated that the average proportion of pigs with mild clinical signs would range from 0.5 – 2.0 percent while 4.0 - 4.5 percent of the herd represents a higher value under routine production. In addition, a morbidity trigger threshold of 9% was suggested as a conservative criterion for an abnormally high proportion of pigs with mild clinical signs (i.e., this morbidity trigger threshold would be expected to have a very low false trigger rate). The predicted time to detection with a morbidity trigger threshold of 9% (mean 20; 95% P.I., 16 – 25 days under the baseline scenario) was shorter compared to the time to detect via daily mortality trigger threshold of 5 per 1000. Even though mild clinical signs are non-specific, they occur earlier in the course of ASFV infection and result in earlier detection at the herd level. Data on severe clinical signs during routine production were not available and hence the false trigger rate could not be calculated for severe clinical signs. However, swine industry subject matter experts opined that one percent of the herd would represent a conservative threshold as severe signs occur at a much lower frequency during routine production.

The time to detection via increased mortality from our study (22, 95% P.I., 17 – 28 days with a daily mortality trigger threshold of 5 per 1000 pigs and baseline contact rate) is similar to Halasa *et al*., 2016 (median 21-28 days); however, the predicted time to detection from our study is substantially longer than the values in Faverjon *et al*., 2020 based on room-level mortality thresholds (8 days) [17, 18]. Possible factors contributing to the relatively shorter time to detection in Faverjon *et al*., 2020 include 1) using highly virulent ASF strain characteristics, 2) starting disease transmission simulations with an infectious pig in contrast with initiating transmission with a latently infected pig as in this study and 3) employing lower trigger thresholds for increased mortality. The infectious period for pigs dying due to ASF for moderately virulent strains of 8.3 (95% P.I.,3.9,14.3) days was substantially longer than that for highly virulent strains used in Faverjon *et al*., 2020. Furthermore, the false trigger frequency was higher in this study due to the greater variability in the mortality data. The mean weekly mortality among the 248 different herds varied considerably (5^th^ and 95^th^ percentiles 0.6 and 6.7 per 1000 respectively). In addition, some herds had a significant positive autocorrelation in the mortality for different weeks at 1 or 2 lags.

Active surveillance via rRT-PCR testing is a key outbreak measure for early detection of ASF. The currently proposed active surveillance protocols for an ASF Control Area are often based on targeted sampling of sick and dead pigs [15]. Several articles report the aggregate clinical score based on the degree of different types of clinical signs to capture the progression of clinical signs [2, 12]. However, it is not straightforward to model the distribution of the clinical score at the herd level to inform simulation models used for surveillance design. The parameters for the time to onset and duration of clinical signs estimated from individual pig level clinical signs are more directly applicable in disease transmission and surveillance models.

There are several alternative approaches to estimate transmission parameters from experimental data. Several studies have used a reconstruction of the transmission process in conjunction with Generalized Linear Models or maximum likelihood estimation to estimate the transmission parameters [8]. However, the reconstruction process requires an important simplifying assumption of deterministic and integer-valued disease state durations. Recently, some studies have used Markov Chain Monte Carlo (MCMC) methods to jointly estimate the disease state durations and the transmission parameters [19]. While this approach has fewer approximations, it necessitates including additional variables for the unobserved disease state transition times and may require a longer computer run time for convergence in some instances [19]. We utilized an acceptance rejection-based approximate Bayesian computation algorithm to estimate the transmission parameter from experimental data. This method enables the consideration of the impact of the variability in infectious state durations and the latent period in the estimation. In addition, the algorithm is easily parallelizable and enables the efficient use of high performance resources.

Although the experimental data used in this study was focused on genotype I moderately virulent ASFV strains, mortality percent for Genotype II moderately virulent strains from Estonia (50%) was in a similar range as the disease mortality percent in the current study (95% P.I., 24% - 57%) [2]. Moreover the time to detection via increased daily mortality is fairly insensitive to the ASF disease mortality percent used as the input in the model. For instance, the mean time to detection only decreased from 22.4 to 20.6 days when disease mortality percent was increased to 90% in an additional scenario. Therefore it is possible that the time to detection for genotype II moderately virulent strains has a range similar to that for genotype I strains in this study, although further evaluation may be necessary.

There are some limitations that must be considered while interpreting the study results. We assumed a constant transmission rate even though the level of shedding is possibly reduced beyond 30 days post infection after the pigs have recovered from the acute infection phase. However, the potentially reduced transmission rate from recovered pigs would arguably have a lesser impact on the time to detection via clinical signs, which would mostly depend on the transmission dynamics during the initial stage of herd infection [20]. For example, the time to detection via increased daily mortality under the fast spread scenario remained virtually unchanged even when the infectious period for pigs that recover was reduced to 25 days in an alternative scenario. Several pigs in de Carvalho Ferreira *et al.*(2013) were intermittently shedding above 1.92 TCID_50_ per swab after recovering from acute ASF infection.

We note that some differences between the field transmission dynamics and the model predictions are to be expected due to factors such as swine health management practices, housing structure, and co-infections that may not be adequately captured by the small-scale, controlled experiments informing the model parameters. The transmission model in this study assumed homogenous transmission within a herd since experimental data used did not include any heterogeneous transmission rates within and between animal subpopulations such as pens or rooms. This may be particularly relevant for large swine operations with multiple barns and rooms within a barn. Evaluating the time to detection for moderately virulent strains using a heterogenous transmission model which incorporates the premises and barn structure is important an area for future study.

In general, the time it takes for producers to identify clinical signs compatible with ASFV infection and then notify veterinarians and regulatory authorities depends on their disease awareness. The level of ASF-awareness likely varies by producer education and proximity to infected farms. The adoption of data based trigger thresholds for disease morbidity and mortality such as those evaluated herein may be beneficial in reducing the variability of the time elapsed prior to initiating further investigation. Given the impact of the above factors, producer outreach regarding the trigger thresholds is critical to increase awareness and their adoption in the field.

We focused on predicting the time interval between herd exposure and notification by the producers to the authorities to initiate further diagnostic investigation in the current study. In the event of an ASF outbreak in the US, movement restrictions would be placed on suspect herds with clinical signs while awaiting diagnostic test results [15]. Such restrictions can reduce further ASF spread from infected premises via movements while waiting for diagnostic test results. However, sample transportation logistics and laboratory capacity may be a limiting factor in countries without strong surveillance systems. Further research regarding the application of syndromic surveillance trigger thresholds together with active surveillance in countries with limited diagnostic testing capacity would be beneficial.

## Conclusion

Understanding ASF transmission dynamics within a herd is important to develop effective outbreak management strategies. In this study, we estimated the transmission model parameters for moderately virulent ASFV strains and predicted the time to detection in finisher swine herds. Our results indicate that it may take two weeks or longer to detect ASF in a finisher swine herd via mild clinical signs or increased mortality beyond levels expected in routine production. A key factor contributing to the extended time to detection is the long latently infected period for an individual pig that results in relatively slower transmission during the initial stages of the herd infection. These transmission model parameter estimates and predicted time to detection provide valuable information to inform emergency preparedness and other simulation models used for evaluating regional disease spread and designing surveillance sampling protocols.

## Data Availability

The data that support the findings of this study are available from Wageningen Bioveterinary Research, the Netherlands but restrictions apply to the availability of these data, which were used under license for the current study, and so are not publicly available. Data are however available from the authors upon reasonable request and with permission of Wageningen Bioveterinary Research.

## Abbreviations

ASF: African Swine Fever
ASFV: African Swine Fever Virus
MCMC: Markov Chain Monte Carlo
rRT-PCR: Real-time Reverse Transcription Polymerase Chain Reaction
qPCR: Quantitative Polymerase Chain Reaction
TCID50: Median Tissue Culture Infectious Dose
